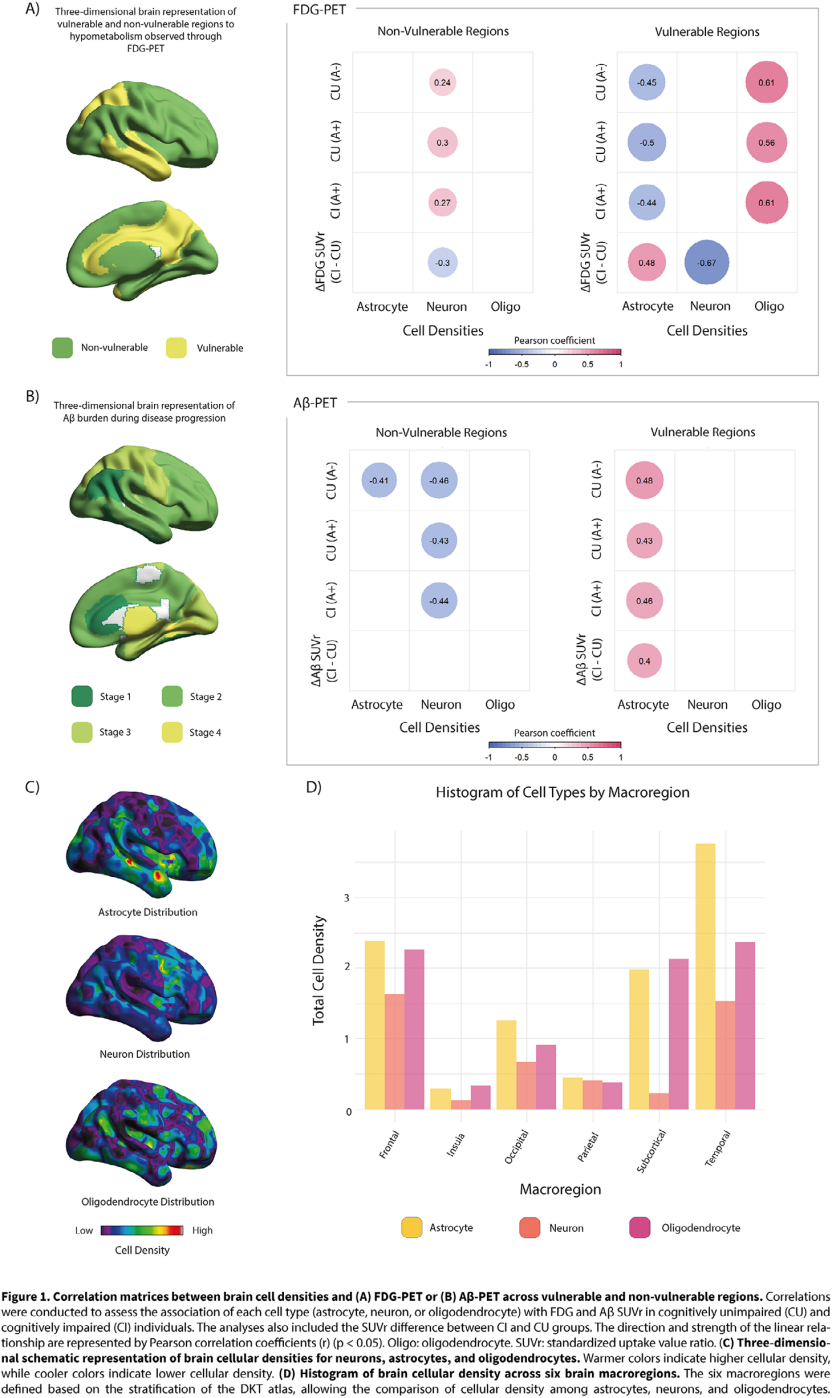# Brain cell densities are associated with hypometabolism and Aβ burden in Alzheimer's disease

**DOI:** 10.1002/alz70856_103086

**Published:** 2025-12-26

**Authors:** Gabriel Colissi Martins, Christian Limberger, Gabriel Lermen Hoffmeister, Mariana Radaelli Schmaedek, Ramon Bertoldi de Souza, Roberta dos Santos de Oliveira, Marco Antônio De Bastiani, Eduardo R. Zimmer

**Affiliations:** ^1^ Universidade Federal do Rio Grande do Sul, Porto Alegre, Rio Grande do Sul, Brazil; ^2^ University of Cologne, Cologne, Germany; ^3^ Universidade Federal do Rio Grande do Sul, Porto Alegre, RS, Brazil; ^4^ McGill University, Montreal, QC, Canada; ^5^ Brain Institute of Rio Grande do Sul ‐ Pontifícia Universidade Católica do Rio Grande do Sul, Porto Alegre, Rio Grande do Sul, Brazil

## Abstract

**Background:**

Neurons and glial cells, such as astrocytes, microglia, and oligodendrocytes, compose and orchestrate the synaptic process. The pathogenesis of Alzheimer's Disease (AD), which includes amyloid‐beta (Aβ) accumulation, can disrupt these cell functions leading to brain glucose hypometabolism, which is generally interpreted as a signal of neurodegeneration. Therefore, it is crucial to understand the potential relationship between neuronal and glial cell densities with AD progression. Here, we investigated how brain cellular densities associate with FDG‐ and Aβ‐PET imaging across regions implicated in AD.

**Method:**

We obtained FDG‐ and Aβ‐PET images from 619 cognitively unimpaired (CU) and impaired (CI) individuals from ADNI. Brain cellular abundance maps were sourced from the Neuropm‐Lab's GitHub repository. Pearson correlations were conducted between cellular densities and the mean FDG or Aβ SUVr across AD vulnerable and non‐vulnerable brain regions (*p* <0.05).

**Result:**

Neuronal density positively correlates with FDG‐PET in non‐vulnerable regions, despite amyloid or cognitive status. However, the delta FDG SUVr (difference between CI and CU) was negatively associated with neuronal density in both non‐vulnerable and vulnerable regions for FDG in A+ individuals. Alternatively, a positive correlation was observed between astrocyte density and delta FDG‐PET SUVr in vulnerable regions. Oligodendrocytes showed a positive correlation in FDG‐vulnerable regions regardless of amyloid or cognitive status (Figure 1A). For Aβ‐PET, astrocyte density presented a negative correlation in CU A‐ individuals in non‐vulnerable regions, while neuronal density presented negative correlations despite amyloid or cognitive status. However, in vulnerable regions astrocytes exhibited positive correlations in all groups including the delta Aβ‐PET SUVr group (Figure 1B).

**Conclusion:**

Our findings reveal that higher astrocyte densities are linked to reduced differences in delta FDG SUVr in AD‐vulnerable regions, whereas neuronal densities show an inverse correlation. Conversely, oligodendrocytes display positive correlations with FDG‐PET. This suggests a compensatory glial metabolism aimed at preserving neuronal homeostasis, supported by the higher proportion of these cells compared to neurons across all brain regions (Figure 1C and D). Additionally, analysis of Aβ‐PET data reveals that regions with a higher astrocyte density are linked to a lower amyloid burden in vulnerable areas, further emphasizing the role of glia on amyloid pathology.